# Evaluating the impacts of stressors of *Pseudomonas syringae* pathovar *tomato* on the effectiveness of multi-locus variable number tandem repeat analysis and multi-locus sequence typing in microbial forensic investigations

**DOI:** 10.1186/2041-2223-5-10

**Published:** 2014-08-07

**Authors:** Mindy James, Ulrich Melcher, Jacqueline Fletcher

**Affiliations:** 1 Department of Entomology and Plant Pathology, Oklahoma State University, Stillwater 74078, OK, USA; 2 Department of Biochemistry and Molecular Biology, Oklahoma State University, Stillwater 74078, OK, USA

**Keywords:** Forensic microbiology, Agricultural biosecurity, Multi-locus variable number tandem repeat analysis, Multi-locus sequence typing, *Pseduomonas syringae* pathovar *tomato*, Bacterial evolution

## Abstract

**Background:**

Crops in the USA are vulnerable to natural and criminal threats because of their widespread cultivation and lack of surveillance, and because of implementation of growing practices such as monoculture. To prepare for investigation and attribution of such events, forensic assays, including determination of molecular profiles, are being adapted for use with plant pathogens. The use of multi-locus variable number tandem repeat (VNTR) analysis (MLVA) and multi-locus sequence typing (MLST) in investigations involving plant pathogens may be problematic because the long lag periods between pathogen introduction and discovery of associated disease may provide enough time for evolution to occur in the regions of the genome employed in each assay. Thus, more information on the stability of the loci employed in these methods is needed.

**Results:**

The MLVA fingerprints and MLST profiles were consistent throughout the experiment, indicating that, using a specific set of primers and conditions, MLVA and MLST typing systems reliably identify *P.s. tomato* DC3000. This information is essential to forensic investigators in interpreting comparisons between MLVA and MLST typing profiles observed in *P.s. tomato* isolates.

**Conclusions:**

Our results indicate that MLVA and MLST typing systems, utilizing the specified primers and conditions, could be employed successfully in forensics investigations involving *P.s. tomato*. Similar experiments should be conducted in the field and with other high-consequence plant pathogens to ensure that the assays are reliable for pathogens infecting plants in their natural environment and for organisms that may display faster rates of mutation*.*

## Background

The American agricultural system is vulnerable to attack by bioterrorists or other criminals in several food-related areas such as production, processing and distribution [1]. Factors increasing the vulnerability of the cropping systems in the US to such attacks include their scattered nature, lack of surveillance, and considerable monoculturing [2].

To prepare for the investigation of possible biological crimes on US agriculture, traditional forensic science techniques are being adapted for use with plant pathogens and other environmental samples that may be associated with agricultural environments [3]. To attribute an agroterrorism or criminal event to a perpetrator, a microbial forensics laboratory often determines a microbial signature or fingerprint for the organism of interest [4,5]. Methods used commonly to fingerprint pathogens, differentiate between microbial strains, and determine microbial relatedness include multi-locus variable number tandem repeat (VNTR) analysis (MLVA) and multi-locus sequence typing (MLST) [4].

VNTRs are short, tandemly repeated genomic sequences, present in the majority of prokaryotic and eukaryotic organisms, which vary in repeat copy number between strains of a single microbial species [6]. Variation in VNTR copy number is often exploited for strain differentiation using MLVA [5]. MLVA typing involves PCR amplification of multiple VNTR loci, followed by electrophoretic separation of the resulting fragments. Variation in the number of repeats at a particular locus results in the production of amplicons of different sizes, creating a VNTR fingerprint for the bacterial strain of interest [7,4]. The fingerprint is then used as a confirmation of microbial species identity. Additionally, hypervariability at a given VNTR locus, which can be used to indicate that different bacterial isolates originated from a common source, may be especially useful for the purposes of attribution [5,8].

MLVA has been used to successfully fingerprint a variety of bacteria, including *Bacillus anthracis, Escherichia coli* O157, *Brucella abortus,* and the plant pathogens *Xylella fastidiosa, Xanthomonas oryzae,* and *Pseudomonas syringae*[9-12]. However, because MLVA relies on genetic loci having intrinsically high mutation rates [9], VNTR loci can be affected by treatments such as environmental stress and serial passaging, leading to alteration in the MLVA fingerprints of organisms of interest [9,13,14]. For this reason, a better understanding of the stability and mutational rates of VNTR loci is needed to permit the interpretation of MLVA results in forensic investigations [15,7].

Forensic investigators may need to identify a suspect microorganism to the strain level. MLST allows for strain-level microbe identification by comparing the sequences of multiple genomic housekeeping genes required for normal functioning of the organism [16,17]. In this method, PCR is used to amplify 450 to 500 bp length fragments of 5 to 10 housekeeping genes. The amplicons are then sequenced and compared with the profiles of isolates stored in searchable databases [16].

MLST has been used effectively to characterize a variety of bacterial species, and has been successfully employed in studies of bacterial recombination and genetic diversity [4,18]. A highly reproducible method, it can be adapted easily to any set of genes by designing specific primers [16,19]. The major strength of MLST lies in its ability to detect recombination; however, the technique does not always provide reliable differentiation of strains from recently evolved bacterial species that display little genetic variability [18,20,17].

The use of common forensic assays, such as MLVA and MLST, may be especially problematic in forensic investigations involving plant pathogens because the long lag periods between the introduction of a pathogen and the discovery of the subsequent disease may provide ample time for the pathogen to undergo evolution in regions of the genome used in the microbe-typing assays [2,21].

*Pseudomonas syringe* pv. *tomato*, used as a model organism by many investigators, is a Gram negative, plant pathogenic bacterium with a worldwide distribution [22,23]. The pathogen infects *Arabidopsis thaliana*, *Brassica* species, and tomato, in the latter of which it causes bacterial speck disease, an economically important disease [24,25]. *P.s. tomato* was chosen for this work because of the availability of several genome sequences and because it meets several criteria for potential bioweapons, including ease of handling, toxin production, rate of infection and spread in nature, and yield losses associated with infection [26].

MLVA and MLST have both been employed by others in the study of *P.s. tomato*. A *P.s. tomato* MLVA assay was developed for rapid strain discrimination and for determining phylogenetic relationships between strains [12]. These and other studies using MLVA to examine the relatedness of *P.s. tomato* strains revealed that the diversity within the pathogen is highly correlated with the host plant species in which the organism lived [12,27]. Similarly, MLST has been used to investigate the genetic stability of *P. syringae* (multiple pathovars) and to resolve the role of recombination in the evolution of the pathogen. Strains of *P. syringae* remained genetically consistent over long periods, indicating that the species is highly clonal [18]. However, using MLST, researchers were able to identify multiple recombination sites within the *P.s. tomato* genome, indicating that recombination contributed greatly to the genetic variation of the organism [28].

In the present study, we examined the ability of MLVA and MLST typing methods to type *P.s. tomato* that was subjected to various treatments, in order to evaluate the appropriateness of their use in microbial forensic investigations involving plant pathogens.

## Methods

### Bacterial strain and experimental treatments

*P.s. tomato* DC3000, originally isolated from infected tomatoes in the Channel Islands, Guernsey, UK, was obtained from the laboratory of Dr Carol Bender, previously of Oklahoma State University. Prior to beginning the experiment, the bacterium was grown in King’s B broth medium under optimum conditions reported for this organism (28°C with shaking at 150 rpm) [29]. This master culture was used in preparation of experimental treatments.

*P.s. tomato* DC3000 was exposed to four treatments, meant to simulate various environmental conditions to which it could be exposed before or during a biological attack, while the bacterium was being sequentially sub-cultured for 1 year to mimic continued growth of the pathogen in the field. Treatments comprised: 1) *P.s. tomato* DC3000 grown under optimum laboratory conditions, 2) *P.s. tomato* DC3000 grown under sub-optimal conditions (nutritional stress), 3) mutagenesis of *P.s. tomato* DC3000 followed by growth under optimum conditions, and 4) *P.s. tomato* DC3000 grown *in planta*. Optimal growth conditions were provided by growth of the bacterium under optimized laboratory conditions [30]. Growth of the organism in nature was simulated by growing it in sub-optimal medium and *in planta*. Mutagenesis of *P.s. tomato* DC3000 was used to discern the effects, if any, of enhanced evolutionary rates on the reliability of the forensic assays.

For treatment 1 (optimum conditions), 40 ml of King’s B (KB) broth was inoculated with 0.1 ml of the *P.s. tomato* master culture and incubated at 28°C with shaking at 150 rpm for 4 days. On day 3 of incubation, 10 ml of the culture was removed, and total genomic DNA was extracted using the Qiagen DNeasy Blood and Tissue Kit in accordance with the manufacturer’s instructions (Qiagen, Valencia, CA, USA). On day 4 of incubation, the remaining culture was used to inoculate fresh King’s B broth as above. This process was repeated every 4 days for 1 year.

For treatment 2 (sub-optimal conditions), 40 ml of mannitol-glutamate broth, a minimal medium, was inoculated with 0.1 ml of the master culture [31]. The culture was incubated at 28°C with shaking as for treatment 1, and DNA extraction and further sub-culturing was carried out as described above.

Mutagenesis was carried out on 10 ml of the *P.s. tomato* master culture using ethyl methanesulfonate (EMS), a chemical that generates mutations by guanine alkylation, following the method described by Thomas and Leary with slight modifications [32,33]. For this, 10 ml of log phase bacteria, in King’s B broth, were exposed to EMS (1 mg/ml of broth) for 4 hours. This culture was diluted 1:20 in fresh medium and incubated at 28°C with shaking at 150 rpm for 24 hours. The bacterial cells were washed by centrifugation and resuspended in fresh King’s B broth [33], then 40 ml of King’s B broth were inoculated with 0.1 ml of the culture. The culture was incubated at 28°C with shaking as with treatments 1 and 2, and DNA extraction and sub-culturing were carried out as described above.

For the *in planta* treatment, 3-week-old tomato (*Lycopersicum esculentum* cv. Glamour) seedlings were inoculated with the master culture of *P.s. tomato* by dipping a sterile swab into a 4-day culture and lightly rubbing the undersides of 3 to 5 expanded leaves. The inoculated plants were maintained in a growth chamber at 25°C with 50% relative humidity and a 12-hour photoperiod. One month after inoculation, leaf tissue was excised from the lesion margins and soaked in 1 ml of sterile water for 3 hours. The resulting suspension was then streaked for isolation on King’s B agar plates, which were incubated at 28°C. When bacterial colonies were obvious, plates were examined using ultraviolet light for the presence of fluorescent colonies typical of *P.s. tomato* grown on this medium [34]. Several fluorescent colonies were transferred to 10 ml of KB broth and incubated at 28°C with shaking at 150 rpm for 24 hours. That bacterial suspension was used to inoculate new tomato seedlings as described above, and the remaining culture was used for DNA extraction as previously described. For this treatment, the *P.s. tomato* culture was transferred seven times over a 10-month period.

DNA samples extracted from liquid cultures at 6-week intervals, and from each culture transfer *in planta*, were subjected to molecular analysis using MLVA and MLST.

### Multi-locus variable number tandem repeat analysis

MLVA analysis of VNTR regions within the *P.s. tomato* genome was carried out using previously described VNTR loci (Table 1), primer pairs (Table 2) and molecular methods [12]. PCR amplification of VNTR loci were performed as singleplex reactions using locus-specific PCR primers (Table 2), GoTaq Flexi DNA Polymerase with accompanying reagents (Promega, Madison, WI, USA), and PCR Nucleotide Mix (Fisher Bioreagents, Pittsburg, PA, USA) in a final reaction volume of 25 μl. Cycling conditions were as follows: 2 minutes at 95°C, followed by 30 cycles of 1 minute at 94°C, 1 minute at 55°C, and 1 minute at 72°C, with a final extension step at 72°C for 7 minutes.

**Table 1:** **Characteristics of VNTR loci**^**a **^**used in MLVA typing of *Pseudomonas syringae pathovar tomato *DC3000**

**VNTR locus**	**Motif length, bp**	**Repeats in Pst DC3000, n**
715	7	7.3
1570	6	13.5
1929	144	1.9
337	125	2.9
919	829	2.0

MLVA, Multi-locus variable number tandem repeat analysis; VNTR, Variable number tandem repeat.

^a^From: Baker [12].

**Table 2:** **VNTR primers used in MLVA typing of *Pseudomonas syringae pathovar tomato *DC3000**

**VNTR locus primer**^**a**^	**Direction**	**Primer sequence (5′ → 3′)**	**Product size, bp**
715-F	Forward	TGTGCGATGACACGCTTACCCATA	314
715-R	Reverse	TATTCGCGGACATTCGTGACAAGA	
1570-F	Forward	AGTCTCTGCTCTTTGGTTGGCGTA	216
1570-R	Reverse	GTCTGATGTACATGGTGCGCTGGT	
1929-F	Forward	CGAACAGAACGCGGCCTTCAAATA	511
1929-R	Reverse	ACAGCGACTGAGCTGATTCAGGAT	
337-F	Forward	TGGAGCACAAACTGCTCTGAGTCT	440
337-R	Reverse	TACAGAGATGGCGCGATTGAGCA	
919/920-F	Forward	AAACATCAGCCAGCAAATCACCCG	829
919/920-R	Reverse	AACTGTTATGCCTTGTCGCACAGC	

MLVA, Multi-locus variable number tandem repeat analysis; VNTR, Variable number tandem repeat.

^a^From: Baker [12].

**Figure 1:**
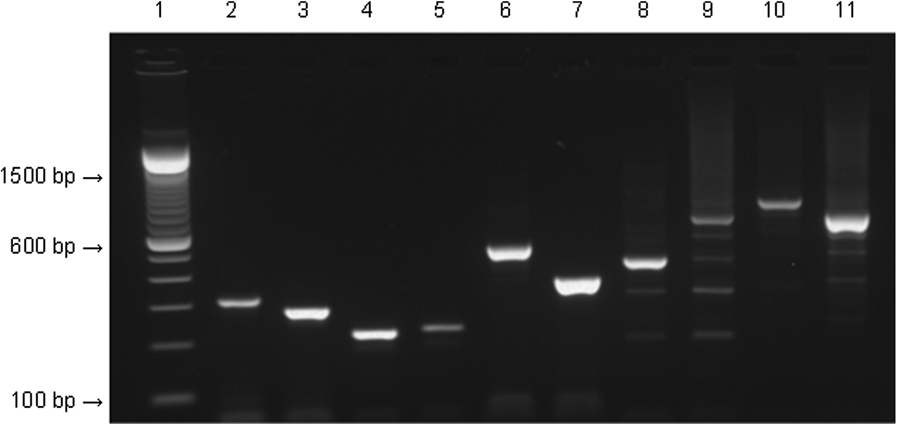
**Size comparison of amplicons resulting from PCR amplification with locus-specific primers used in *Pseudomonas syringae pathovar tomato *DC3000 multi-locus variable number tandem repeat analysis (MLVA) typing assay.** Lane 1, 100 bp DNA ladder; lane 2, locus 715 of *P.s. tomato* DC3000; lane 3, locus 715 of *P.s. tomato* 1318; lane 4, locus 1570 of *P.s. tomato* DC3000; lane 5, locus 1570 of *P.s. tomato* 1318; lane 6, locus 1929 of *P.s. tomato* DC3000; lane 7, locus 1929 of *P.s. tomato* 1318; lane 8, locus 337 of *P.s. tomato* DC3000; lane 9, locus 337 of *P s. tomato* 1318; lane 10, locus 919 of *P.s. tomato* DC3000; lane 11, locus 919 of *P.s. tomato* 1318.

Following amplification, the MLVA fingerprint for each sample was visualized by gel electrophoresis using a 1.5% agarose gel supplemented with 0.1 μl/ml of SyBR®Safe DNA Gel Stain (Invitrogen, Carlsbad, CA, USA). To ensure that electrophoresis could adequately distinguish between the amplicon sizes, MLVA was also performed on a separate *P.s. tomato* strain, *P.s. tomato* 1318, having different numbers of repeats from *P.s. tomato* DC3000 at three of the chosen VNTR loci: *P.s. tomato* 1318 has one fewer repeat at loci 715 and 1929, and four additional repeats at locus 337 than *P.s. tomato* DC3000. A 100 bp DNA ladder (Invitrogen) was used with all gels. The sizes of the resulting amplicons were representative of the number of repeats at each locus in *P.s. tomato* 1318, and could be distinguished easily from those in *P.s. tomato* DC3000, based on size (Figure 1).

### Multi-locus sequence typing

MLST analysis of the *P.s. tomato* genome was carried out using previously published genes, primers, and molecular methods [18,35]. The core genome components evaluated encode for glyceraldehyde-3-phosphate dehydrogenase (*gapA*), phosphofructokinase (*pfk*), sigma factor 70 (*rpoD*), aconitate hydratase B (*acnB*), phosphoglucoisomerase (*pgi*), gyrase (*gyrB*), and citrate synthase (*cts*).

PCR amplification of each gene was carried out on 10 ng of template DNA using gene-specific PCR primers (Table 3), GoTaq Flexi DNA Polymerase and accompanying reagents (Promega, Madison, WI), and PCR Nucleotide Mix (Fisher Bioreagents, Pittsburg, PA) in a final reaction volume of 25 μl. Cycling conditions were as follows: 2 minutes at 94°C, followed by 30 cycles of 1 minute at the appropriate annealing temperature (Table 4), and 1 minute at 72°C. Following this initial PCR reaction, the PCR products were cleaned using ExoSAP-IT PCR cleanup reagent (Affymetrix, Santa Clara, CA, USA) in accordance with manufacturer’s instructions. The clean products were then employed as template in a second amplification reaction as preparation for sequencing.

**Table 3:** **Primers used in multi-locus sequence typing of *Pseduomonas syringae *pathovar *tomato*** DC3000

**Primer**^**a**^	**Direction**	**Sequence (5′ → 3′)**
acn-Fp^b^	Forward	ACATCCCGCTGCACGCYCTGGCC
acn-Rp^b^	Reverse	GTGGTGTCCTGGGAACCGACGGTG
acn-Fs^b^	Forward	ATGAARCAGATMGAAGAAATGCGCGG
acn-Rs^b^	Reverse	GCCRACCATCTTYTGCGCMAGGG
cts-Fp^b^	Forward	AGTTGATCATCGAGGGCGCWGCC
cts-Rp^b^	Reverse	TGATCGGTTTGATCTCGCACGG
cts-Fs^b^	Forward	CCCGTCGAGCTGCCAATWCTGA
cts-Rs^b^	Reverse	ATCTCGCACGGSGTRTTGAACATC
gapA-Fps^b^	Forward	CGCCATYCGCAACCCG
gapA-Rps^b^	Reverse	CCCAYTCGTTGTCGTACCA
gyrB-Fps^c^	Forward	MGGCGGYAAGTTCGATGACAAYTC
gyrB-Rps^c^	Reverse	TRATVKCAGTCARACCTTCRCGSGC
pfk-Fp^b^	Forward	ACCMTGAACCCKGCGCTGGA
pfk-Rp^b^	Reverse	ATRCCGAAVCCGAHCTGGGT
pfk-Fs^b^	Forward	AGCAAYATCAAGMTGGCCGA
pfk-Rs^b^	Reverse	ACCATGCCKGCCARMAGCG
pgi-Fp^b^	Forward	TCAAGGACTTCAGCATGCGCGAAGC
pgi-Rp^b^	Reverse	CGAGCCGCCCTGSGCCAGGTACCAG
pgi-Fs^b^	Forward	TTCAGCATGCGCGAAGCG
pgi-Rs^b^	Reverse	TGCGCCAAGGTACCAGG
rpoD-Fp^c^	Forward	AAGGCGARATCGAAATCGCCAAGCG
rpoD-Rps^c^	Reverse	GGAACWKGCGCAGGAAGTCGGCACG
rpoD-Fs^b^	Forward	AAGCGAATCGAAGAAGGCATYCGTG

^a^p-PCR primer, s-sequencing primer.

^b^Primers from: Sarker and Guttman [18].

^c^Primers from: Sawada *et al*. [35].

**Table 4:** ***Pseduomonas syringae *pathovar *tomato *DC3000 multi-locus sequence typing PCR primer annealing temperatures**

**PCR primer set**	**T**_**a**_**,°C**^**a**^
acn	60
cts	56
gapA	62
gyrB	63
pfk	63
pgi	60
rpoD	63

^a^T_a_, annealing temperature.

For the sequencing reaction, a master mix was prepared for each primer, consisting of 10 μl sterile water, 3 μl 5× Sequencing Buffer (BigDye Terminator v1.1/3.1; Applied Biosystems Carlsbad, CA, USA), 2 μl 10 mM individual primer (Table 3), 2 μl Ready Reaction Mix (BigDye v3.1; Applied Biosystems), and 2 μl of cleaned PCR product from each sample. Cycling conditions were as follows: 30 seconds at 96°C, followed by 26 cycles of 15 seconds at 50°C, and 4 minutes at 60°C. Prior to sequencing, ethanol precipitation was performed on each PCR product, then 12 μl of sterile water, 5 μl 3 M ammonium acetate and 57 μl of 100% ethanol were added to each sample, and mixed before centrifugation at 1,500 *g* for 30 minutes. After discarding the supernatant, 70 μl of 70% ethanol was added to each sample, and tubes were centrifuged at 1,500 *g* for 15 minutes. The supernatant was discarded, 10 μl of deionized water was added to the pellet, and tubes were mixed to suspend the DNA. The DNA was sequenced by the Oklahoma State University Recombinant DNA/Protein Core facility using a DNA analyzer (ABI Model 3730; Applied Biosystems). The resulting DNA sequences were aligned, trimmed, and analyzed using MEGA 4: Molecular Evolutionary Genetics Analysis software [36].

## Results

### Multi-locus variable number tandem repeat analysis

**Figure 2:**
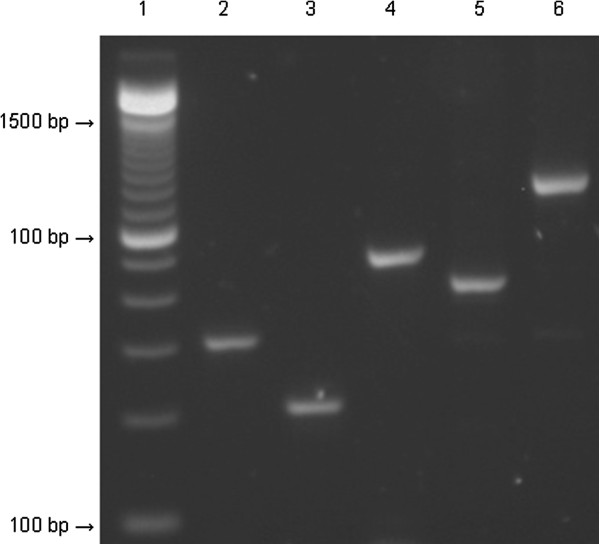
**Representative multi-locus variable number tandem repeat analysis fingerprint for *Pseudomonas syringae *pathovar *tomato *DC3000.** Lane 1, 100 bp DNA ladder; lane 2, locus 715; lane 3, locus 1570; lane 4, locus 1929; lane 5, locus 337; lane 6, locus 919.

**Figure 3:**
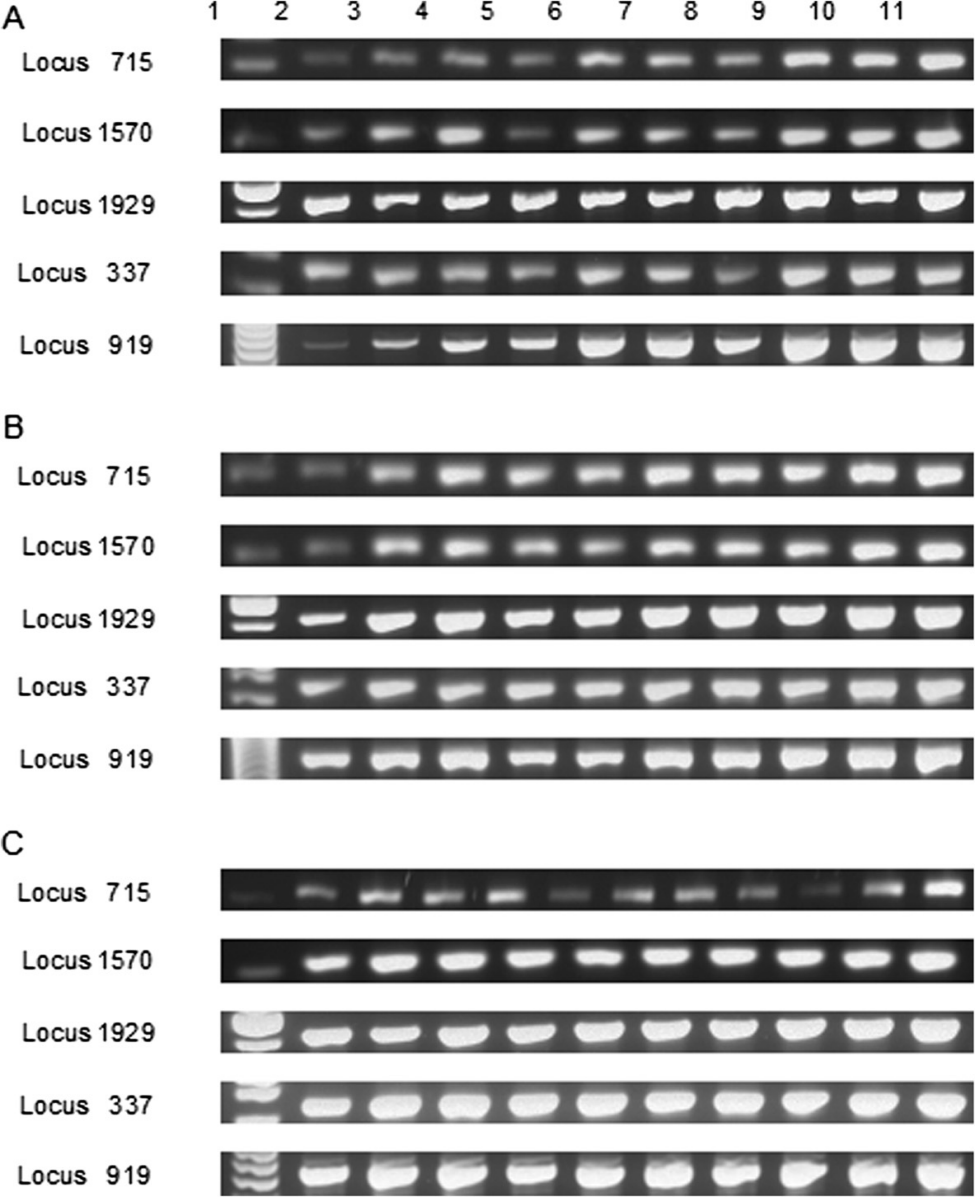
**Comparison of agarose gel analysis of multi-locus variable number tandem repeat analysis performed on *Pseudomonas syringae *pathovar *tomato *DC3000 exposed to various experimental treatments. (A) ***P.s. tomato* DC3000 exposed to optimum growth conditions; **(B) ***P.s. tomato* DC3000 exposed to sub-optimal growth conditions; **(C)** mutagenized *P.s. tomato* DC3000 exposed to optimum growth conditions. Lane 1, 100 bp DNA ladder; lane 2, original *P.s. tomato* DC3000 culture used in treatment preparation; lane 3, sub-culture 11; lane 4, sub-culture 22; lane 5, sub-culture 33; lane 6, sub-culture 44; lane 7, sub-culture 55; lane 8, sub-culture 66; lane 9, sub-culture 77; lane 10, sub-culture 88, lane 11; sub-culture 92.

**Figure 4:**
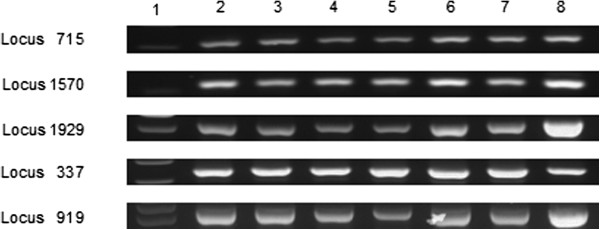
**Comparison of agarose gel analysis of multi-locus variable number tandem repeat analysis performed on *Pseudomonas syringae *pathovar *tomato *DC3000 after passage through tomato.** Lane 1, 100 bp DNA ladder; lane 2, passage 1; lane 3, passage 2; lane 4, passage 3; lane 5, passage 4; lane 6, passage 5; lane 7, passage 6; lane 8, passage 7.

The MLVA typing system employed in this project utilized primers specific for five known VNTR loci within the *P.s. tomato* genome (Table 1). MLVA typing of the master *P.s. tomato* DC3000 culture used to inoculate the experimental treatments resulted in a baseline fingerprint for the organism (Figure 2), in which the amplicon size for each primer pair was as expected (Table 2). MLVA fingerprints obtained for sub-cultures 11, 22, 33, 44, 55, 66, 77, 88, and 92 of non-mutagenized and mutagenized *P.s. tomato* DC3000 grown under optimal or sub-optimal conditions and *P.s. tomato* DC3000 from each plant passage were consistent with the baseline fingerprint of the organism, and did not appear to change over time. To ensure that no repeats were gained or lost over time, PCR products from amplification of each locus for all samples from each treatment were compared by gel electrophoresis. All bands for each locus were of the correct size (Table 2) and appeared to be, within the resolution of our analysis technique, indistinguishable for all samples (Figure 3, Figure 4).

### Multi-locus sequence typing

A MLST typing system targeting seven core genome components of *P.s. tomato* was employed in this study (Table 3). Sequences for each gene were aligned and trimmed to a consistent length (Table 5). Analysis of the trimmed gene sequences from the master culture of *P.s. tomato* DC3000, sub-cultures 11, 22, 33, 44, 55, 66, 77, 88, and 92 of non-mutagenized and mutagenized *P.s. tomato* DC3000 grown under optimal or sub-optimal conditions, and *P.s. tomato* DC3000 from each plant passage revealed no mutations during the sampling period.

**Table 5:** **Trimmed sequence lengths for genes used in *P.s. tomato *MLST assay**

**Gene**	**Gene length, bp**^**a**^	**Trimmed sequence length, bp**
Acn	399	477
Cts	445	478
GapA	497	625
GyrB	480	575
Pfk	414	619
Pgi	448	576
RpoD	452	546

^a^From: Sarkar and Guttman [18].

## Discussion

Attribution of a biocrime or bioterror event involving a plant pathogen may require a forensics laboratory to determine a microbial fingerprint or profile for the organism [4,5]. Previous research has shown that VNTR loci may undergo mutation in response to serial passaging and environmental stressors such as increased temperature, starvation, and irradiation [14,13]. For example, *E. coli* O157:H7 grown with creek water as a sole nutrient source underwent triple and quadruple repeat changes within VNTR loci [13]. Similarly, *B. abortus* strain 544 was shown to gain a repeat in three observed VNTR loci during serial passaging; however, three other strains of the pathogen showed no change [14]. In the current study, the MLVA fingerprint for *P.s. tomato* DC3000, generated using primers for five specific VNTR loci (Table 2), did not change over time, and was not affected by the experimental treatments. These results indicate that the VNTR regions employed in the *Pst* MLVA assay are stable within the genome and are not affected by culturing conditions, including growth in the plant host. Thus, the assay could reliably type the organism in an investigation involving the pathogen; however, similar experiments should be performed in the field to ensure that other adverse natural conditions will have no effect on the validity of the assay. These findings could also be of use for investigation of natural events and in epidemiological studies.

Our MLST results correspond with the previous findings that the core genome of *P. syringae* is highly clonal and displays very little genetic heterogeneity [18]. Each passage consisted of taking 0.1 ml of culture into 40 ml of medium and incubating for 4 days. This is a 400× increase in the number of cells per culture period, or approximately 10 generations per sub-culture. With a total length of 2.3 kbp for the MLST products and an expectation of 1 × 10^−8^ substitutions/bp/generation, we would anticipate approximately 0.023 substitutions. By observing nearly 100 samples per experiment and including a treatment that should have dramatically increased the mutation rate, we expected to see a change in the target sequences. However, the nucleotide sequences of the seven housekeeping genes employed in the *P.s. tomato* MLST assay did not change over time, and were not affected by the experimental treatments, indicating that MLST could also be employed successfully in an investigation involving the pathogen. As MLST typing systems are particularly useful for detection of recombination, the results of the *P.s. tomato* MLST assay could be affected by the presence of other microorganisms during growth of the pathogen [18,20,28]. To further ensure the validity of the assay, similar experiments should be performed in the field under natural environmental conditions and in the presence of a variety of other microbes.

The MLVA and MLST assays employed in this experiment did not reveal any differences in the DNA sequences assessed, based on the various culturing conditions tested. We were somewhat surprised at this result, as a variety of treatments were continued for a full year; however, selection of different VNTR loci or housekeeping genes might reveal changes (not observed in our work) within the *P.s. tomato* DC3000 genome. In a follow-up, collaborative study currently underway, whole genome sequencing of these strains will provide a more comprehensive view of genetic changes in these and other strains of *P.s. tomato.*

The fact that, in our experiment, both MLVA and MLST typing systems were effective for characterization of *P.s. tomato* in microbial forensic investigations does not mean that all phytopathogenic bacterial species will have similarly low mutation rates. Plant pathogens belong to a variety of taxonomic kingdoms and genera, and infect various plant hosts in many different environments, and it is likely that these factors will influence the specific mutation rate of each organism. Experiments similar to those conducted here should be carried out with other important plant pathogens to ensure the validity of MLVA and MLST typing systems for those organisms. In new pathogens, whole genome sequencing could be employed to identify stable VNTR loci and housekeeping genes within the genome that could be employed in development of MLVA and MLST assays for the pathogen of interest.

## Conclusions

The results of this study indicate that MLVA and MLST typing systems, utilizing the specified primers and conditions, could be employed successfully in forensic investigations involving *P.s. tomato*. However, similar experiments should be conducted in the field and with high-consequence plant pathogens to ensure that the assays are reliable for organisms infecting plants in nature, and for use with other plant pathogens that may display faster rates of mutation than *P.s. tomato*.

## Abbreviations

EMS: Ethyl methanesulfonate; MEGA: Molecular Evolutionary Genetics Analysis; mlMLST: Multi-locus sequence typing; MLVA: Multilocus variable number tandem repeat analysis; pv: Pathovar; T_a_: Annealing temperature; VNTR: Variable number tandem repeat.

## Competing interests

The authors declare that they have no competing interests.

## Authors’ contributions

MJ participated in the design of the study and carried out the experimental design, bacterial typing, data analysis, and drafted the manuscript. UM conceived of the study, participated in the design of the study and data analysis, and was involved in drafting of the manuscript. JF participated in design of the study and data analysis, and was involved in drafting of the manuscript. All authors read and approved the final version of the manuscript.
